# Do Not BASILICA the New Valve

**DOI:** 10.1016/j.jaccas.2025.103912

**Published:** 2025-06-04

**Authors:** Jonathan X. Fang, Pedro A. Villablanca

**Affiliations:** a Center for Structural Heart Disease, Henry Ford Health System, Detroit, Michigan, USA; b National Heart Centre Singapore, Singapore

**Keywords:** aortic valve, complication, valve replacement

## Abstract

**Object:**

Bioprosthetic or native Aortic Scallop Intentional Laceration to prevent Iatrogenic Coronary Artery obstruction (BASILICA) is currently the standard technique for leaflet modification in transcatheter aortic valve replacement to overcome coronary obstruction. The technique involves traversal of the coronary cusp with a 0.014-inch coronary wire, snaring the wire, and then lacerating the leaflet with a denuded portion of the wire known as the flying V. The procedure requires careful planning and technical expertise. We report a new complication of BASILICA where prepositioning of a transcatheter heart valve (THV) before laceration accidentally damaged the THV.

**Key Steps:**

This complication originates from the inadvertent crossing of the straight-tip wire between the flying V and the aortic cusp when the flying V was not well apposed to the cusp. Prepositioning of the THV has become a variation of the procedure for patients with a high perceived risk of hemodynamic instability after BASILICA. This backfired, resulting in a complicated procedure.

**Potential Pitfalls:**

The exact position of the 0.014-inch wire and flying V might be difficult to visualize in patients with a large body habitus. Operators should check that the flying V is at the cusp level before crossing the aortic valve with a wire. During the initial delivery of the flying V to the cusp, care should be taken to avoid losing the wire in the guide. Multicenter data of BASILICA show that hemodynamic instability is uncommon in single-leaflet lacerations. Therefore, prepositioning of a THV before leaflet laceration is usually unnecessary and requires strong clinical justification if pursued

**Take-Home Messages:**

BASILICA is a complex procedure. Operator familiarity with potential pitfalls and appropriate bailout methods are necessary. Operators should ensure that the flying V is well apposed to the leaflet before crossing into the left ventricle with a wire to prepare for valve deployment.

## Visual Summary

**Figure:**
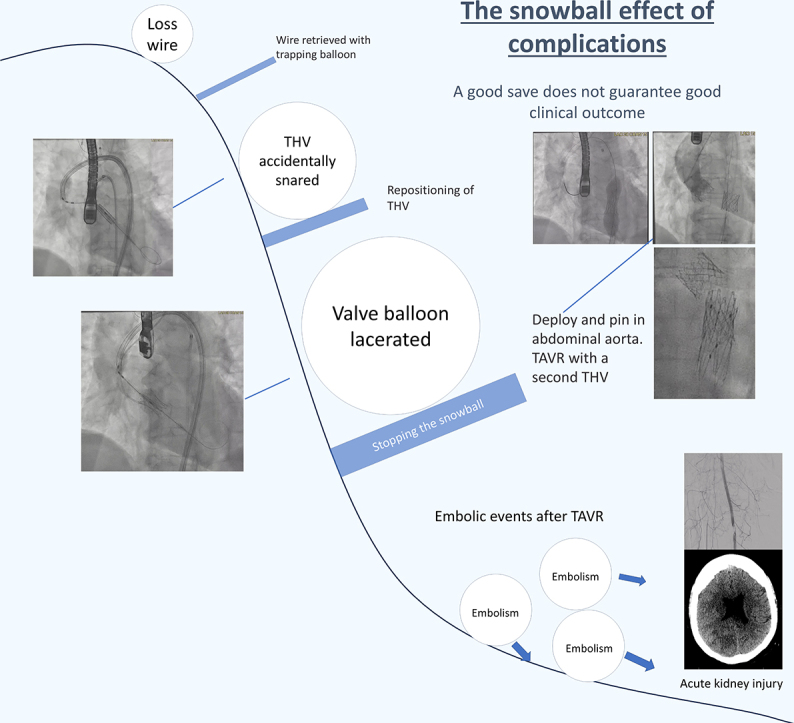

The Bioprosthetic or native Aortic Scallop Intentional Laceration to prevent Iatrogenic Coronary Artery obstruction (BASILICA) during the transcatheter aortic valve replacement (TAVR) procedure^1^ is currently the standard technique for leaflet modification during TAVR to prevent coronary artery obstruction. The procedure involves traversal of the coronary cusp with a coronary wire under electrosurgery cut, followed by snaring of the wire and then laceration of the leaflet with a denuded and kinked portion of the coronary wire, known as the flying V, under electrosurgery cut. The flying V is delivered to the coronary cusp by feeding the wire through 1 guide and pulling the snared wire through another guide. Potential pitfalls include traversal failure, persistent coronary obstruction when the traversal is not basal enough or the anatomy is unfavorable for BASILICA, or inadvertent damage to surrounding structures. Anatomical factors favoring BASILICA include a prosthetic valve leaflet that extends above the coronary ostia in systole and a short valve-to-coronary distance of <4 mm in valves with reasonable commissural alignment and a low coronary height of 10 mm in patients with native valves. Additional considerations include sinus width <30 mm and a short valve frame to sinotubular junction distance of <3 mm. Clinical factors favoring BASILICA over coronary protection and chimney stenting include long life expectancy and anticipation of coronary revascularization in the future.

**Take-Home Message**

• Operators should ensure that the flying V is well apposed to the leaflet before crossing into the left ventricle with a wire to prepare for valve deployment.

## Case Summary

**Figure 1:**
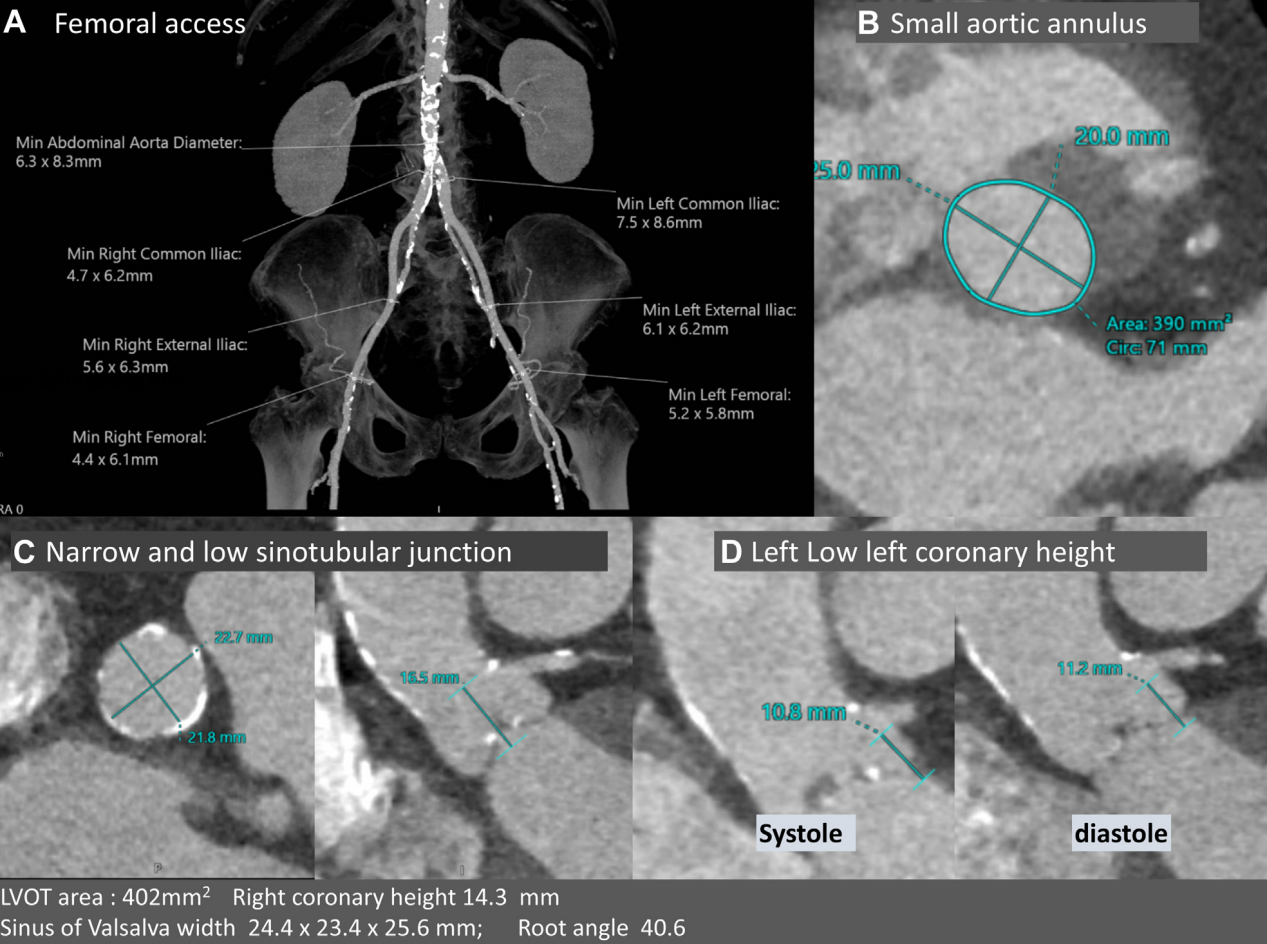
Access and Computed Tomography Workup (A) Adequate femoral access. (B to D) Annulus, sinotubular junction, and left coronary height. LVOT = left ventricular outflow ract.

**Figure 2:**
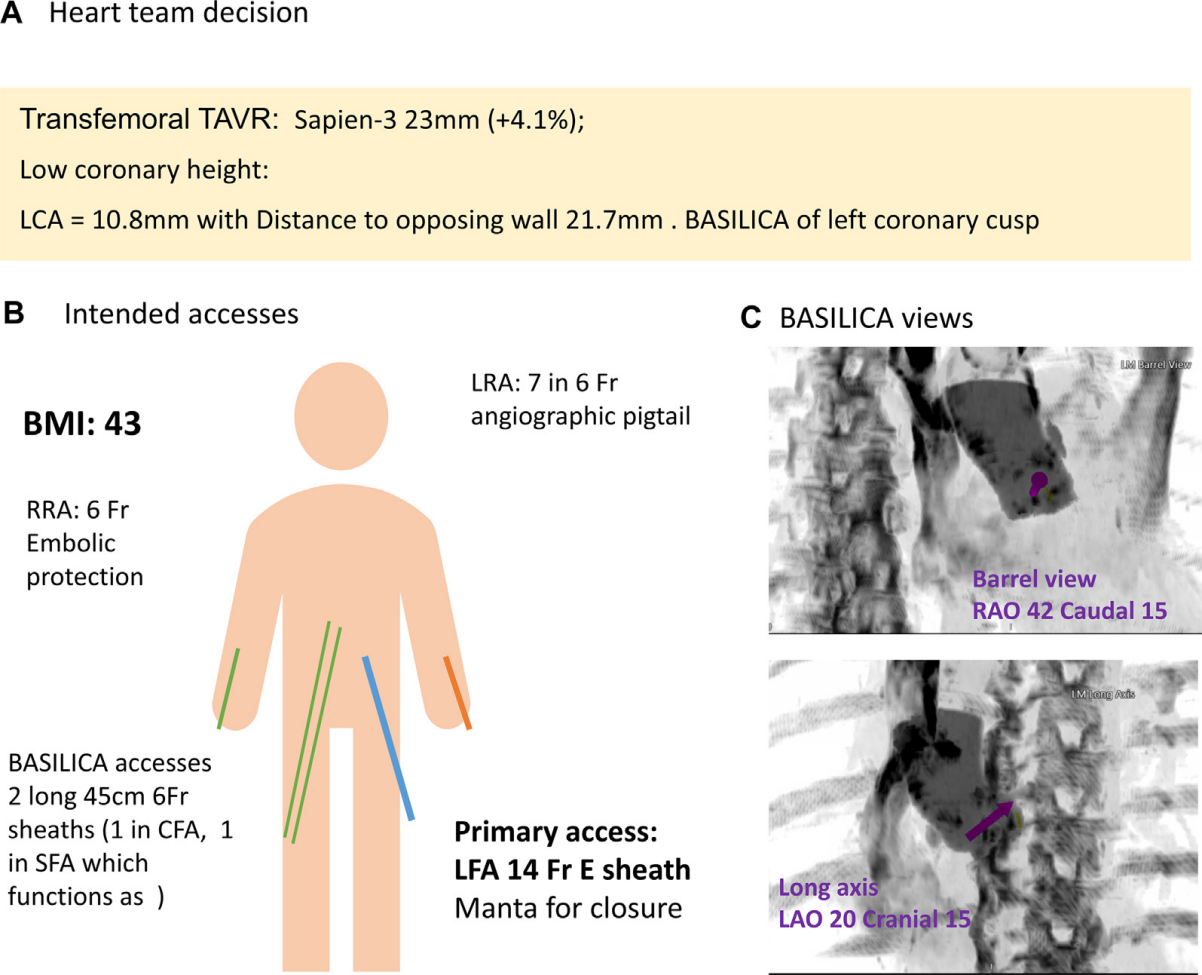
Heart Team Plan and BASILICA Angles Two 45-cm 6-F sheaths were used for BASILICA. Embolic protection was used from radial access. BASILICA = Bioprosthetic or native Aortic Scallop Intentional Laceration to prevent Iatrogenic Coronary Artery obstruction; BMI = body mass index; CFA = common femoral artery; LAO = left anterior oblique; LCA = left coronary artery; LFA = left common femoral artery; LRA = left radial artery; RAO = right anterior oblique; RRA = right radial artery; SFA = superficial femoral artery; TAVR = transcatheter aortic valve replacement.

A 71-year-old woman presented with symptomatic aortic stenosis. She had a body mass index of 43 kg/m^2^, multivessel non-bstructive coronary artery disease up to 60%, and stage 3b chronic kidney disease with an estimated glomerular filtration rate of 35 mL/min/1.73 m^2^. Her Society of Cardiothoracic Surgeons Predicted Risk of Operative Mortality was 5.0%, and surgical evaluation concluded that she had a very high surgical risk. Echocardiography revealed an aortic valve area of 0.81 cm^2^, peak velocity of 3.75 m/s, gradient of 56.1/27.7 mm Hg, and stroke volume index of 29.91 mL/m^2^ with mild regurgitation. Her femoral arteries were favorable for access. She had an aortic annulus area of 390 mm^2^, a low coronary height of 10.8 mm for the left main coronary artery, a distance to the opposing wall of 21.7 mm, estimating valve-to coronary distance of 2.7 mm, sinus width of 24.4 mm, and a low and narrow sinotubular junction (Figure 1). Transfemoral access was feasible. A heart team meeting opted for TAVR with a balloon-expandable 23-mm valve using a left femoral access with leaflet modification using BASILICA because of the risk of coronary obstruction and anticipation of future need of percutaneous coronary intervention (Figure 2).

**Figure 3:**
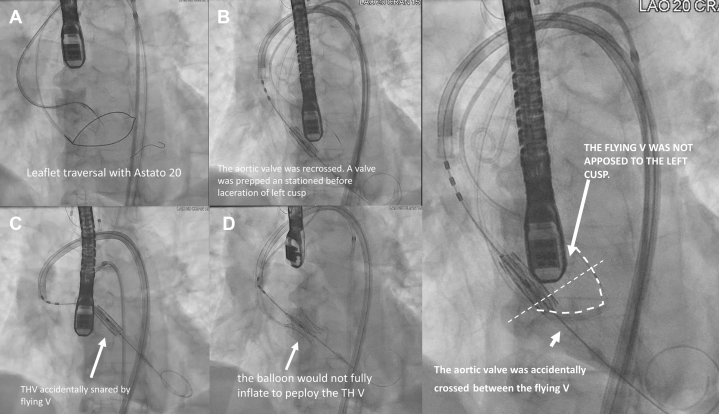
Accidental Snaring of the THV (A) Leaflet traversal. (B) Positioning the transcatheter heart valve (THV). (C and D) THV accidentally snared by the flying V and lacerating the balloon. (E) Position of the flying V was identified to be ventricular.

**Figure 4:**
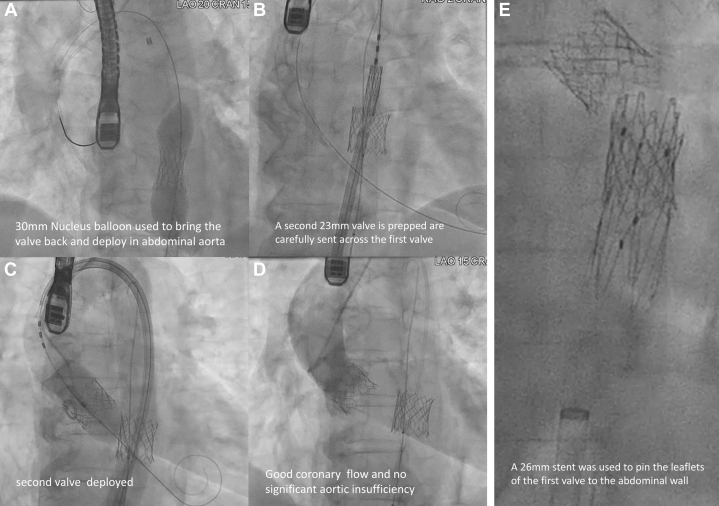
Aortic Deployment of the First THV and Transcatheter Aortic Valve Replacement (A) A nucleus balloon brought back the first transcatheter aortic valve (THV) to the descending aorta. (B to D) Transcatheter aortic valve replacement with a second THV. (E) A stent was used to pin the leaflet of the first THV to the aorta.

We performed BASILICA with leaflet traversal in the standard manner. However, during the delivery of the flying V, the snared wire was lost in a 45-cm-long sheath in the abdominal aorta, requiring removal by balloon trapping against the sheath followed by en-bloc removal of the sheath and reinsertion. Subsequently, we crossed the valve with a straight-tip wire from the primary access, placed a pigtail catheter, exchanged it for a circular-tip wire, and prepared a 23-mm balloon-expandable transcatheter heart valve (THV). We prepositioned the THV just above the annular level before lacerating the left cusp to shorten the time between laceration and valve deployment. However, subsequent leaflet laceration accidentally snared the THV (Video 1) because of inadvertent wire crossing between the aortic cusp and the flying V as the flying V was not well apposed to the cusp. Regardless, transesophageal echocardiography confirmed laceration of the left cusp and no evidence of damage to intracardiac structures. We therefore proceeded to reposition the THV across the annulus for deployment. However, the balloon of the THV was found to be lacerated and could not be inflated to deploy the THV (Figure 3). We pulled the valve back and deployed it in descending aorta and took a new 23-mm balloon-expandable valve for TAVR and deployed it while paying attention to the low sinotubular junction height, with a good result (Figure 4). A cerebral embolic protection device was used. During this process from leaflet laceration to deployment of a second valve, the patient remained hemodynamically stable.

**Figure 5:**
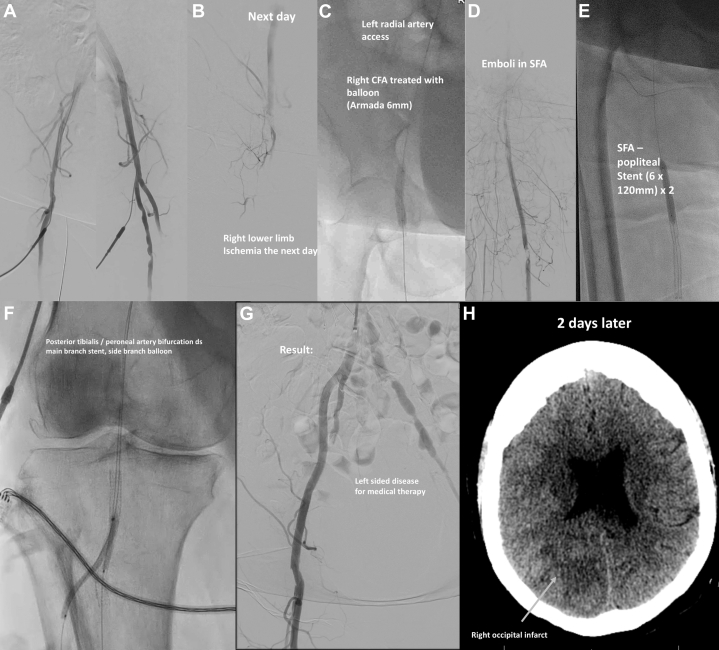
Acute Lower Limb Ischemia and Stroke From Multiple Emboli (A) Angiograms before closure. (B) Right femoral angiogram the next day. (C to G) Further emboli and disease downstream requiring revascularization. (H) Right occipital infarct after 2 days. Abbreviation as in Figure 2.

The patient was stable and asymptomatic on the first day postprocedure. However, the patient subsequently experienced embolic phenomena, presenting with acute right lower limb ischemia the next day. Angiography showed occlusion at the level of the common femoral artery with poor distal runoff, which we managed with balloon angioplasty through the left radial approach. Interprocedurally, additional emboli to the superficial femoral artery and pre-existing critical disease in the posterior tibialis-peroneal bifurcation required further revascularization from a right femoral access. We achieved a good angiographic result, but 2 days later the patient presented with acute stroke with left homonymous hemianopia and was deemed unsuitable for cerebral reperfusion therapy because of high bleeding and procedural risk. Computed tomography showed a right occipital infarct (Figure 5). Three days later, she developed acute kidney injury that responded poorly to dialysis. She was transitioned to comfort care and died 10 days afterward.

**Figure 6:**
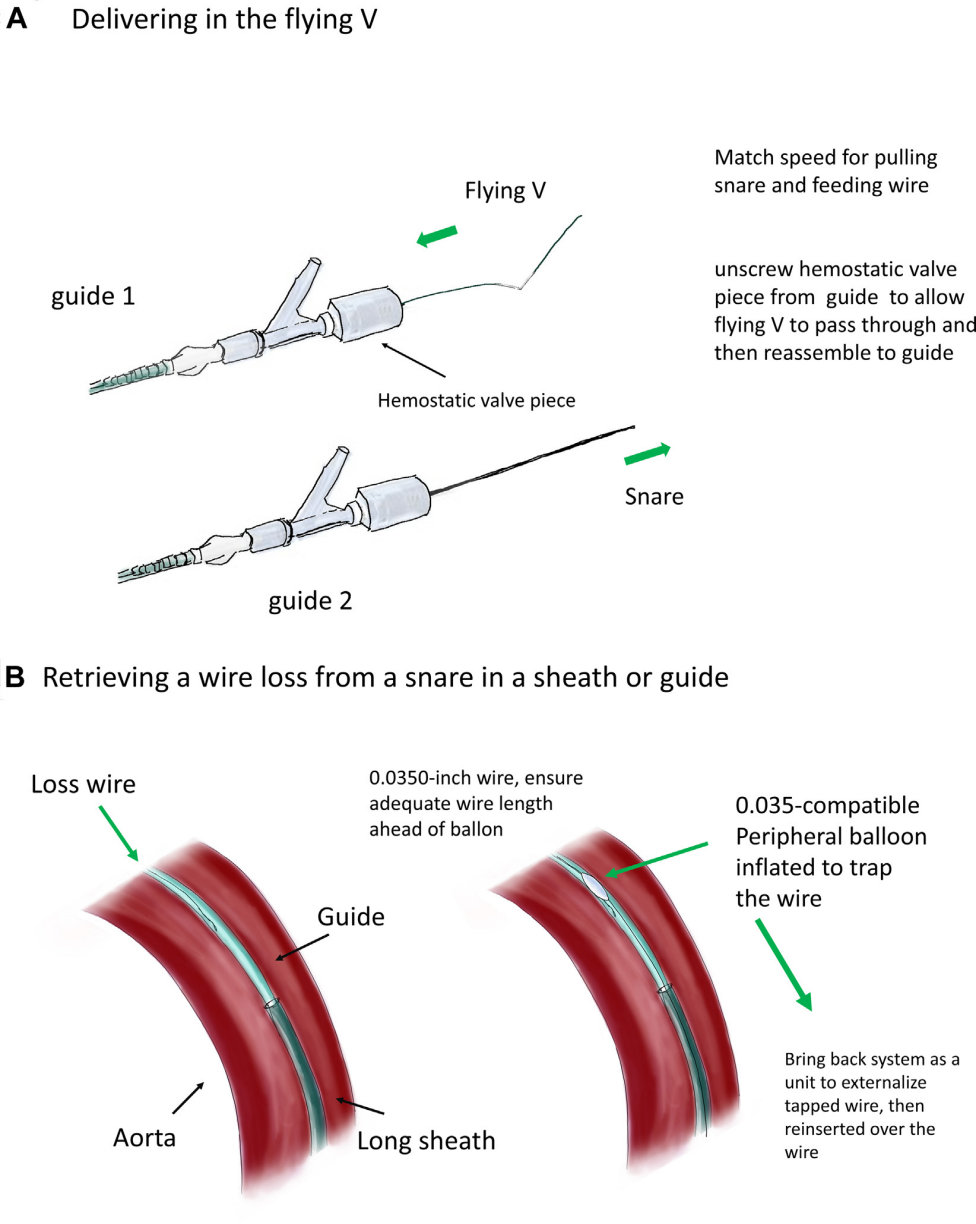
Schematic Diagram (A) Flying V delivery. (B) Balloon-trapping technique for wire rescue.

**Visual Summary:**
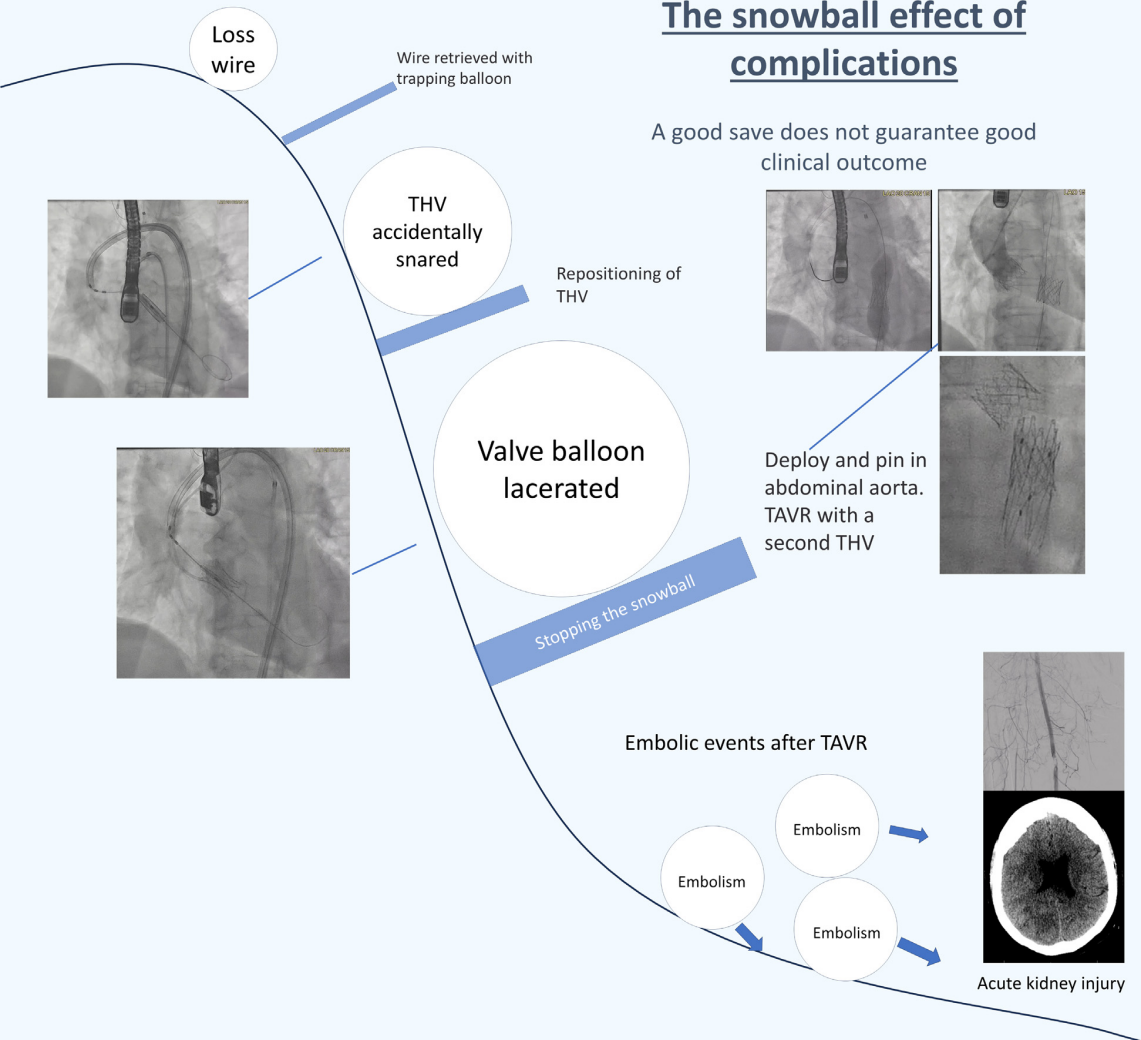
The Snowball Effect of Complications THV = transcatheter aortic valve; other abbreviation as in Figure 2.

## Pitfalls and Corrective Procedural Steps

1. Retrieval of a lost wire from a snare (Figure 6)
  ○ BASILICA requires 2 guiding catheters, 1 for traversal and 1 for snaring. After leaflet traversal with a 0.014-inch coronary wire, usually the Astato 20 wire (Asahi Intecc Medical, USA), under electrosurgery cut, the wire is snared by a gooseneck snare. The wire is then carefully brought back into the snaring guide, whereas a flying V is created and fed through the hemostatic valve system of the traversal guide. To negotiate the flying V through the hemostatic valve, we recommend removing the valve from the guide, manually negotiating the flying V through it, and then reattaching the valve to the guide. The speed of feeding the wire should match that of pulling the snare; otherwise, the wire could be lost. This is especially important when the feeding and pulling of the wire are performed by 2 operators.
  ○ If the wire is lost inside the guide or the long sheath, a 0.035-inch compatible peripheral balloon can be advanced to the position of the lost wire and inflated against the guide or sheath to trap the wire. Then, the guide and sheath can be removed as a unit to externalize the lost wire. Adequate wire length should be ensured ahead of the balloon to avoid losing access. The sheath with a dilator is then carefully reinserted over both wires. Alternatively, multiple coronary guidewires can be passed to the point of wire loss and intertwined by rotation to manually create a snaring system suitable for use in a confined space. The intertwined wires can then be pulled back as a unit and untwined once the loss wire is externalized.
2. How the accidental snaring of the valve could have been avoided
  ○ After BASILICA, severe aortic regurgitation may occur. Before laceration, the aortic valve is usually crossed to place a pigtail catheter in the left ventricle. The leaflet is then lacerated, and a circular-tip wire is placed in the ventricle through the pigtail to deliver a prepared THV for deployment.
  ○ In this case, we prepositioned a THV above the aortic annulus to shorten the time with severe aortic regurgitation. However, this led to inadvertent damage of the THV by the flying V.
  ○ From the multicenter perspective BASILICA trial, transient hypotension is uncommon, occurring in 6.7% of cases during BASILICA (2/30).^1^ In vitro studies have also shown that after BASILICA without balloon augmentation, the leaflet splay tends to fold back in diastole. Therefore, strong clinical justification is required for propositioning of THV near the annulus before leaflet laceration when performing BASILICA.
  ○ Operators should check the position of the flying V and ensure that it is well apposed to the base of the cusp before crossing into the left ventricle.
3. How to deploy a balloon-expandable THV when its balloon is ruptured
  ○ Wire access to the valve should always be maintained.
  ○ The Nucleus balloon (B. Braun Interventional Systems, USA) has a waist and can be used to retrieve undeployed THVs by gentle inflation within the THV and deployment in the descending aorta away from side branches.
  ○ Avoid inflation of valves or balloons that are oversized compared with aortic diameter, as doing so could lead to aortic dissection.
  ○ A stent can be used to pin the leaflets of the THV to the wall to avoid potentially detrimental hemodynamic effects of pin-wheeled leaflets of valves deployed in aortas smaller than the valve.
4. Snowball effect of complications
  ○ Complications could result from minor errors. Even with appropriate management, sequelae such as embolic phenomenon, which may or may not be related, could still occur

## Conclusions

BASILICA is a complex procedure that requires a systematic approach to ensure procedural success. The flying V position should be checked for good apposition to the cusp before recrossing into the left ventricle with any wire. Hypotension during BASILICA is uncommon. Therefore, prepositioning of a THV during BASILICA is usually unnecessary and should be used only when strongly justified. Embolic phenomenon might occur after TAVR with BASILICA.

## Funding Support and Author Disclosures

Dr Villablanca is a consultant for Edwards Lifesciences and Teleflex. Dr Fang has reported that he has no relationships relevant to the contents of this paper to disclose.

## Appendix

Video 1 Procedural steps, including the accidental snaring of the transcatheter aortic valve (0:12).

## References

[1] Khan J.M., Greenbaum A.B., Babaliaros V.C. (2019). The BASILICA trial: prospective multicenter investigation of intentional leaflet laceration to prevent TAVR coronary obstruction. JACC Cardiovasc Interv.

